# The combination of Qing Kailing injection and clindamycin injection leads to systemic adverse reactions: Case report

**DOI:** 10.1097/MD.0000000000040657

**Published:** 2024-11-22

**Authors:** Shenghui Zhou, Shouhui Wang, Yajun Wang, Fenglin Xi

**Affiliations:** a Department of Pharmacy, Baiyin Central Hospital, Baiyin, Gansu, China.

**Keywords:** clindamycin, drug–drug interactions, Qing Kailing injection, systemic adverse reactions

## Abstract

**Rationale:**

It is imperative to be cautious about the potential systemic allergic reaction caused by the combined use of Qing Kailing Injection (QKI) and clindamycin as it may be life-threatening.

**Patient concerns:**

A 48-year-old female with a history of hypertension was admitted to a private hospital with a fever and cough. She was diagnosed with lung infection and received QKI infusion, followed by clindamycin infusion. During clindamycin infusion, the patient experienced convulsions followed by nausea and vomiting (gastric contents). Upon arrival at the emergency room, she lost consciousness, became agitated and sweaty, and had incontinence. Respiratory rate was measured at 32 breaths per minute, pulse rate was 180 beats per minute, temperature was 42°C, and blood pressure was unmeasurable.

**Diagnosis:**

Drug hypersensitivity reactions.

**Interventions:**

The patient immediately discontinued clindamycin, administered dexamethasone 10 mg intravenously, and was subsequently referred to our hospital for further treatment. Emergency physicians immediately provided oxygen inhalation via a face mask, administered midazolam for sedation and ibuprofen combined with physical measures for cooling, carried out fluid replacement, administered furosemide for diuresis, and avoided using allergenic drugs.

**Outcomes:**

The patient regained consciousness, without experiencing further convulsions, nausea, vomiting, fever, or abnormal urination, and their vital signs stabilized.

**Lessons:**

Caution should be exercised in the concurrent use of traditional Chinese medicine injections and antibacterial drugs in clinical treatment because of the potential for allergic reactions to both medications. If combination therapy is deemed necessary, adequate flushing of the infusion pipe with saline or replacement with a new infusion device is recommended, while closely monitoring the patient for any signs of allergic reactions.

## 1. Introduction

During treatment, patients often receive multiple medications simultaneously, particularly when dealing with the different types or clinical manifestations of the disease. Antimicrobials are commonly used to address infectious disorders by inhibiting or killing microbial growth. Chinese herbal injection (CHI), a product that combines traditional Chinese medicine with modern technology, has demonstrated high bioavailability and efficacy in the treatment of various diseases. However, some CHIs were withdrawn from the National Health Commission of China because of serious adverse events. Here, we present a case of systemic adverse reactions caused by a combination of CHI and antimicrobials for the treatment of a pulmonary infection in a patient with hypertension.

## 2. Case report

A 48-year-old Asian female was admitted to the hospital with convulsions, fever, nausea, vomiting of the gastric contents, unconsciousness, restlessness, sweating, and incontinence, all occurring within the past hour. She reported the onset of fever and dry cough 3 days earlier. She had previously sought treatment at a private hospital where she was diagnosed with pulmonary infection. At 12:00 pm, she received a 20 mL infusion of Qing Kailing injection (QKI; Hebei Shenwei Pharmaceutical Co., Ltd) diluted in 200 mL of a 10% glucose injection. Thirty-five minutes later, at 12:35 pm, clindamycin (0.3 g) diluted in 100 mL of 0.9% sodium chloride solution was administered. Approximately 8 minutes into the clindamycin infusion (at 12:43 pm), the patient experienced convulsions and fever, with a recorded temperature of 39.2°C; no rash was observed. The patient had a history of penicillin allergy, hypertension, and allergic purpura, and was not compliant with antihypertensive medications, resulting in poor blood pressure control. There was no family history of hereditary diseases.

Physical examination revealed that the patient was in a state of altered consciousness and had experienced persistent convulsions. A cranial examination revealed no deformities, tenderness, or masses. The patient exhibited an acute febrile appearance and profuse sweating, with normal skin and mucous membranes and no rash. Abdominal examination results were unremarkable and renal assessment results were normal. Vital signs included a respiratory rate of 32 breaths per minute, a pulse rate of 180 beats per minute, and a temperature of 42°C, while blood pressure was unmeasurable.

Laboratory tests revealed the following results: white blood cell count of 3.51 × 10^9^/L (reference range: 4–10 × 10^9^/L), neutrophils at 76.3% (reference range: 50–70%), hemoglobin level of 101 g/L (reference range: 113–172 g/L), platelet count of 75 × 10^9^/L (reference range: 85–320 × 10^9^/L), amylase level of 40 U/L (reference range: <140 U/L), and calcium ion level of 2.45 mmol/L (reference range: 2.1–2.7 mmol/L). The convulsions were not attributed to hypocalcemic seizures, and all other indicators were within the normal limits. A head CT scan revealed no significant abnormalities, effectively ruling out brain diseases, such as epilepsy.

**Figure 1.**
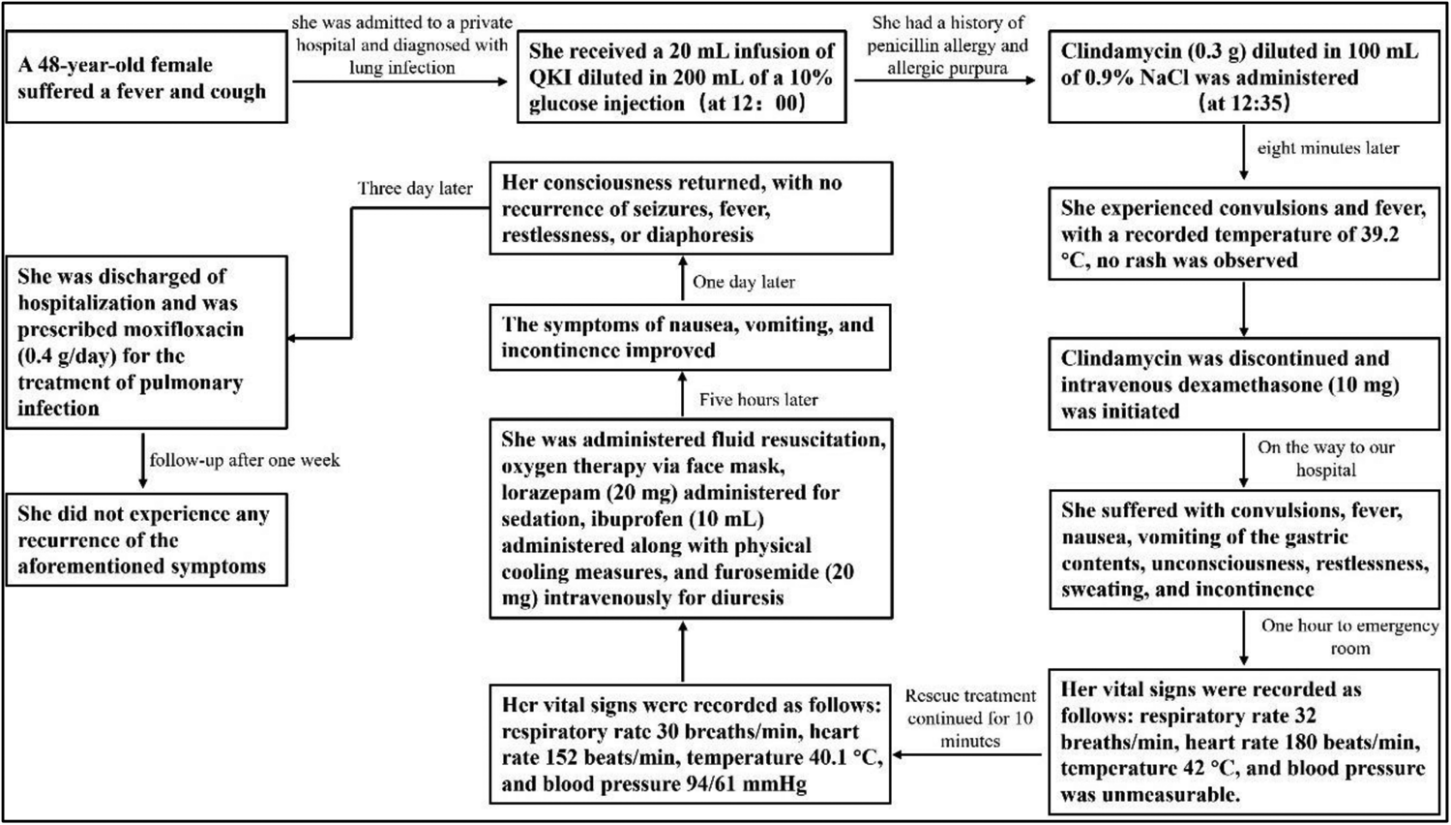
The timeline of this case. QKI = Qing Kailing injection.

The patient was diagnosed with drug hypersensitivity reactions based on the physical examination and medication history. All medications were discontinued at a private hospital and intravenous dexamethasone (10 mg) was initiated. Upon arrival at our emergency room, physicians administered resuscitation treatment, including intravenous access for fluid resuscitation, oxygen therapy via face mask, lorazepam (20 mg) administered via a micro-infusion pump at a rate of 4 mL/h for sedation, ibuprofen (10 mL) administered when the temperature exceeded 38.5°C along with physical cooling measures, and furosemide (20 mg) intravenously for diuresis. Allergenic drugs were avoided due to the patient’s highly sensitive state. Ten minutes later, her vital signs were recorded as follows: respiratory rate 30 breaths per minute, heart rate 152 beats per minute, temperature 40.1°C, and blood pressure 94/61 mm Hg. The symptoms of nausea, vomiting, and incontinence improved 5 hours later. Consciousness returned within 1 day, with no recurrence of seizures, fever, restlessness, or diaphoresis. The patient was discharged after 3 days of hospitalization and was prescribed moxifloxacin (0.4 g/day) for the treatment of pulmonary infection. During follow-up after 1 week, the patient did not experience any recurrence of the aforementioned symptoms. The timeline of this patient is shown in Figure 1.

This case report was approved by the Research Ethics Committee of Baiyin Central Hospital and written informed consent was obtained from the patient for publication.

## 3. Analysis and discussion

Naranjo’s score scale, proposed by the Canadian physicist Naranjo in the 1980s,^[1,2]^ categorizes the causal link between adverse drug reactions (ADR) and drugs into 4 categories: “definite,” “probable,” “possible,” and “doubtful.” A score of 9 to 10 points was defined as “definitely” that caused the ADR, which was confirmed using objective evidence and quantitative assays. A sum of 5 to 8 “probably” caused the ADR, which was supported by objective evidence and quantitative assays. A sum of 1 to 4 “possibly” caused the ADR, and the data could not be confirmed or discredited. A score of <1 indicated that an association with the drug was “doubtful,” with a lack of association or chance.

QKI, renowned for its heat-clearing, detoxifying, phlegm-eliminating, and collateral-unblocking properties, constitutes a blend of 8 traditional Chinese medicinal herbs, namely *cholalic acid*, *Concha margaritifera*, *hyodeoxycholic acid*, *Fructus Gardeniae*, *Cornu Bubali*, *Radix Isatidis*, *Baicalin*, and *Flos Lonicerae*. Clinically, it is primarily used in the treatment of acute and chronic hepatitis, upper respiratory tract infections, pneumonia, and cerebrovascular disorders.^[3,4]^ In November 2001, the China Food and Drug Administration (CFDA) reported the first case of anaphylaxis attributed to QKI. In recent years, the CFDA has continued to receive notifications of severe ADRs associated with QKI, notably those involving nervous system damage, manifested as coma, convulsions, and lethargy. In this case, the patient received 20 mL of QKI diluted with 200 mL of 10% glucose for a pulmonary infection, strictly adhering to the prescribed medication and dosage. According to the Pharmacopoeia of the People’s Republic of China (2020), 10% glucose injection, with a pH range of 3.2 to 6.5, is mildly acidic, and it has been documented that the pH can further decrease to 6.12 upon admixture with other drugs.^[5]^ However, as outlined in Table 1, a total score of 3 points signifies a “probable” causal relationship between these ADRs and QKI usage. This may be because QKI is prone to precipitation in acidic environments, leading to adverse reactions. Our hypothesis attributes this primarily to *cholalic acid* and *flavonoids* present in QKI.^[6]^ Hence, compatibility issues may lead to precipitation and subsequent ADRs. The recommendation is to dilute QKI with saline to prevent precipitation and administer it slowly, followed by careful observation of allergic reactions within 30 minutes.^[7]^ In adults, the drip rate of the drug solution should be controlled within a range of 40 to 60 droplets per minute,^[8]^ and the patient’s response should be closely monitored during this critical period.

**Table 1:** Results of Naranjo’s score of adverse reactions caused by QKI.

Relevant issues	Score			
	Yes	No	Do not know	Reasons for scoring
1	+1			The instruction mentions the above adverse reactions
2	+2			The above adverse reactions occurred after the use of QKI
3	+1			The adverse reactions were alleviated within 1–2 days after drug withdrawal
4			0	The patient did not take the drug again after discontinuation
5	−1			The adverse reactions can be induced by clindamycin according to the instruction and literature
6			0	The patient did not take any placebo
7			0	Undetermined
8			0	Unknow
9			0	Unknow
10		0		No objective evidence can confirm the reaction
Total score	3			

QKI = Qing Kailing injection.

By binding to the 50S subunit of sensitive bacterial ribosomes and disrupting protein synthesis, clindamycin is effective in treating infections caused by Gram-positive bacteria.^[9]^ However, it is accompanied by several well-documented side effects, including gastrointestinal distress, manifesting as nausea, vomiting, diarrhea, and pseudomembranous enteritis.^[10–12]^ Additionally, clindamycin has been reported to induce skin reactions,^[13–16]^ anaphylactic shock,^[17–21]^ and convulsions.^[22]^ In this case, the patient received clindamycin for a pulmonary infection, and the final concentration was less than the recommended concentration of 6 mg/mL. The patient exhibited higher fever and convulsions during clindamycin infusion. She experienced nausea, vomiting of the gastric contents, unconsciousness, restlessness, sweating, and incontinence on the way to our hospital. Clindamycin also scored 3 points on the Naranjo scale, indicating a “probable” causal relationship (Table 2). In this study, both the QKI and clindamycin received a score of 3 on the Naranjo scale, signifying a “possible” causal relationship. It is crucial to consider the possibility that the adverse reactions observed may have been triggered by drug–drug interactions.

**Table 2:** Results of Naranjo’s score of adverse reactions caused by clindamycin.

Relevant issues	Score			
	Yes	No	Do not know	Reasons for scoring
1	+1			The instruction and literature mention the above adverse reactions
2	+2			The adverse reactions happened during the infusion of the clindamycin
3	+1			The adverse reactions were alleviated within 1–2 days after drug withdrawal
4			0	The patient did not take the drug again after discontinuation
5	−1			The adverse reactions can be caused by QKI according to the instruction
6			0	The patient did not take any placebo
7			0	Undetermined
8			0	Unknow
9			0	Unknow
10		0		No objective evidence can confirm the reaction
Total score	3			

There is a substantial body of literature on the confluence of traditional Chinese medicine injections and antimicrobial agents, the combination of which may lead to incongruities and potentially induce adverse effects. Zhu^[23]^ observed clindamycin solidifying like jelly upon contact with Potassium Sodium Dehydroandrographolide Succinate for injection (PDSI). A study on the compatibility stability of PDSI and 8 types of antibiotics indicated altered characteristics, precipitation, and formation of a cloudy solution following PDSI combined with clindamycin.^[24]^ Jin and Chen^[25]^ noted the appearance of yellow flocculent precipitates in the filter during intravenous drip infusion of clindamycin and Tanreqing injection. It has been found that the combination of clindamycin and Xueshuantong in compatibility stability studies can lead to increased hemolysis.^[26]^ Nan and Liao^[27]^ reported a case in which hematuria occurred when clindamycin was combined with Riboflavin Sodium Phosphate injection. The co-administration of QKI with certain antimicrobial drugs, such as Cefotaxime, Lincomycin, Ceftriaxone, Cefazolin, Levofloxacin, Azithromycin, Penicillin, Gentamycin, Amikacin, has been reported to be contraindicated owing to compatibility issues, and in some cases, it can lead to patient fatality.^[28]^

The CFDA and Ministry of Health and State Administration of Traditional Chinese Medicine jointly issued the Basic Principles of Chinese Herbal Injections for clinical use.^[29]^ It stipulates that CHI should be used alone and caution should be exercised when combined with other medications. If necessary, to combine with other medicines, attention should be paid to the interval duration and potential drug–drug interactions. QKI is prone to precipitation when encountering an acidic solution. Therefore, after discontinuation of the QKI infusion, the infusion line should be flushed with over 50 mL of saline or the infusion device should be replaced to avoid adverse effects caused by the mixing and precipitation of the 2 drugs inside the pipeline. In the present case, the interval between the clindamycin and QKI infusions was excessively brief, and neither flushing of the infusion line with saline nor replacement with a new infusion device was performed. This nonstandardized conduct is likely due to the inadequate operational capacity of the hospital staff. To prevent similar incidents from occurring and to ensure patient medication safety, we recommend both theoretical and practical training to effectively enhance the operational capacity of community staff through continual theoretical learning, routine assessments, hands-on training, and ongoing education.

Considering the literature, the Naranjo score, and case analysis in this study, we argue that the potential for adverse reactions in this patient was more likely to be associated with QKI-clindamycin interactions.

## 4. Conclusion

In contrast to traditional herbal medicine dosage forms, CHI possesses heightened bioavailability and rapid onset of action, making it irreplaceable in the treatment of acutely critical illnesses. However, owing to the complex chemical composition of CHI, its physicochemical environment can be easily influenced when combined with other medications, thereby altering the stability of major pharmacodynamic components and potentially resulting in ADRs. If co-administration with other therapeutics is necessary in clinical practice, it is advisable to flush the infusion pipeline with over 50 mL of saline or replace the infusion device with another drug infusion before and after administration. Furthermore, close observation of patient responses within 30 minutes of infusion is imperative. Early identification of adverse effects enables the prompt implementation of corresponding therapeutic interventions or rescue measures.

## Author contributions

**Writing – original draft:** Shenghui Zhou.

**Writing – review & editing:** Shouhui Wang, Yajun Wang, Fenglin Xi.

## References

[1] Naranjo CA Busto U Sellers EM. A method for estimating the probability of adverse drug reactions. Clin Pharmacol Ther. 1981;30:239–45. 7249508 10.1038/clpt.1981.154

[2] Naranjo CA Shear NH Lanctôt KL. Advances in the diagnosis of adverse drug reactions. J Clin Pharmacol. 1992;32:897–904. 1447396 10.1002/j.1552-4604.1992.tb04635.x

[3] Yan SK Xin WF Luo GA Wang YM Cheng YY. Simultaneous determination of major bioactive components in Qingkailing injection by high-performance liquid chromatography with evaporative light scattering detection. Chem Pharm Bull. 2005;53:1392–5. 10.1248/cpb.53.1392 16272719

[4] Committee of Chinese Pharmacopoeia. Chinese Pharmacopoeia. Peking: Chemical Industry Press; 2020.

[5] Xie JP Jing HJ Zhang WB Meng W. An Expedition of feasibility on Qingkailing injection with compatibility of commonly used antibiotics, usual vitamin. Chin Pharm Aff. 1999;13:127.

[6] Zhou BX Xi XM. Hematuria induced by Qing-Kai-Ling injection intravenous drip: a case report. Chin J Gen Pract. 2009;7:973–4.

[7] Wang R. Rational application of Qingkailing injection. Henan Tradit Chin Med. 2015;15:2552–4.

[8] Xiang HY. Insights into the adverse effects and clinical rational application of Qingkailing injection. Chin J Exp Tradit Med Formulae. 2010;16:199–200.

[9] Smeets TJ Jessurun N Härmark L Kardaun SH. Clindamycin-induced acute generalised exanthematous pustulosis: five cases and a review of the literature. Neth J Med. 2016;74:421–8. 27966434

[10] Xu RX. Case of hiccups caused by clindamycin. J New Med. 2012;43:140–51.

[11] Ye LP Li DG. Clindamycin phosphate-induced severe hiccups: a case report. Eval Anal Drug Use Hosp China. 2016;16:1584.

[12] Zheng QF Dong HF. Clindamycin and sodium chloride injection induced pseudomembranous enteritis: a case report. Mod J Integr Tradit Chin West Med. 2011;20:3864–5.

[13] Yang DL Jiang ML Dai M. A case of Clindamycin phosphate induced generalized bullous drug eruption in a puerperal woman. Chin J Clin Ration Drug Use. 2020;13:136–7.

[14] Wang LX. Ambulance experience about severe anaphylactic reactions induced by Clindamycin phosphate intravenous drip. Chin J School Doctor. 2015;29:946–8.

[15] Zhu DJ. A case of clindamycin caused exfoliative dermatitis. Chin Foreign Med Res. 2012;10:57.

[16] Ren YJ Xiao B Ye YL. Two cases of clindamycin epidermolysis bullosa drug eruption. Herald Med. 2012;31:1233.

[17] Liu XJ. Anaphylactic shock caused by clindamycin intravenous drip: a case report. Chin J School Doctor. 2014;28:616.

[18] Wei W Gao XT Huang YY Liu H. Anaphylactic shock caused by clindamycin phosphate: a case report. Chin J Pharmacoepidemiol. 2015;24:492.

[19] Wang F. Anaphylactic shock induced by clindamycin injection: a case report. Chin J Clin Ration Drug Use. 2014;7:10–9.

[20] Chen JR. Anaphylactic shock induced by clindamycin phosphate: a case report. Pharm J Chin Peoples Liberation Army. 2016;32:288.

[21] Jin MH. Anaphylactic shock caused by clindamycin phosphate: a case report. Chin Community Doctors. 2012;14:283.

[22] Zhang XL Cui M. Three cases of clindamycin-induced convulsions. Chin J New Drugs. 2003;06:453.

[23] Zhu Z. The presence of the combination taboo in potassium sodium dehydroandrographolide Succinate and clindamycin phosphate. Chin Nurs Res. 2007;31:2905.

[24] Gan QX. The compatibility stability study of Chinese medicine injections (CMI) and antibiotics. Asia Pacific Tradit Med. 2013;9:40–1.

[25] Jin YA Chen J. The presence of the Combination taboo in Tanreqing injection and clindamycin injection. Shandong Med J. 2009;49:76.

[26] Lv LY Liu GP Zhang ML Luo Y. The compatibility stability study of Xeshuantong injection and clindamycin injection. Sci Tech Dev Enterp. 2018;10:97–9.

[27] Nan L Liao SM. Two cases of clindamycin combined with Riboflavin Sodium phosphate injection lead to hematuria. Med J National Defending Forces Southwest China. 2009;19:686.

[28] Li H Deng J Yue Z Zhang Y Sun H. Detecting drug-herbal interaction using a spontaneous reporting system database: an example with benzylpenicillin and qingkailing injection. Eur J Clin Pharmacol. 2015;71:1139–45. 26159784 10.1007/s00228-015-1898-8

[29] National Medical Products Administration Notification on further strengthening the production and clinical use management of traditional Chinese medicine injections. 2008. https://www.nmpa.gov.cn/xxgk/fgwj/qita/20081224120001195.html. Accessed 2023.

